# Molecular Features for Probing Small Amphiphilic Molecules with Self-Assembled Monolayer-Protected Nanoparticles

**DOI:** 10.1021/acs.langmuir.9b03686

**Published:** 2020-04-29

**Authors:** Domenico Marson, Zbyšek Posel, Paola Posocco

**Affiliations:** † Department of Engineering and Architecture, University of Trieste, 34127 Trieste, Italy; ‡ Department of Informatics, Jan Evangelista Purkyně University, 40096 Ústí nad Labem, Czech Republic

## Abstract

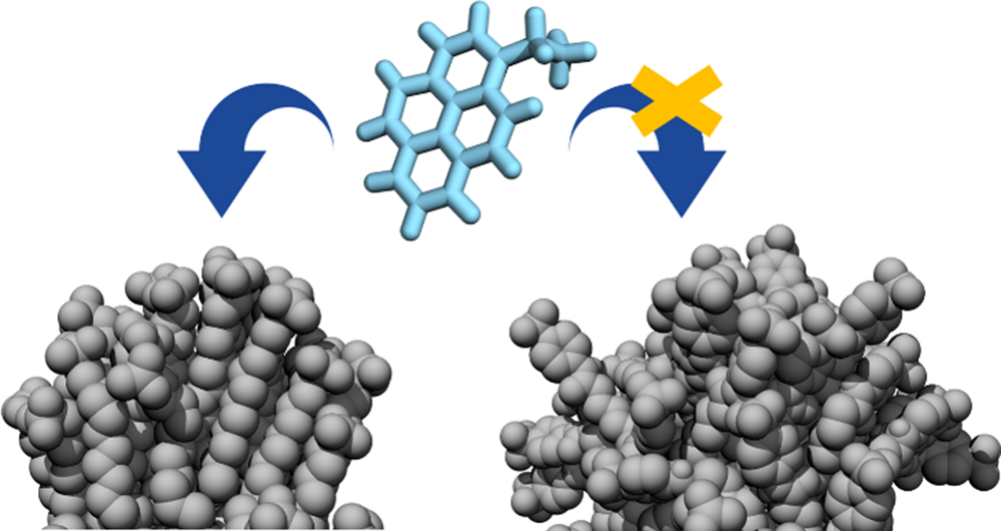

The sensing of small molecules poses the challenge of developing devices able to discriminate between compounds that may be structurally very similar. Here, attention has been paid to the use of self-assembled monolayer (SAM)-protected gold nanoparticles since they enable a modular approach to tune single-molecule affinity and selectivity simply by changing functional moieties (i.e., covering ligands), along with multivalent molecular recognition. To date, the discovery of monolayers suitable for a specific molecular target has relied on trial-and-error approaches, with ligand chemistry being the main criterion used to modulate selectivity and sensitivity. By using molecular dynamics, we showcase that either individual molecular characteristics and/or collective features such as ligand flexibility, monolayer organization, ligand local ordering, and interfacial solvent properties can also be exploited conveniently. The knowledge of the molecular mechanisms that drive the recognition of small molecules on SAM-covered nanoparticles will critically expand our ability to manipulate and control such supramolecular systems.

## Introduction

Sensitive, selective chemical and biological sensors are highly demanded in a broad range of applications in chemistry, biology, healthcare, medicine, and environmental protection. Nevertheless, the development of more efficient, low-cost, versatile, and miniaturized sensors requires continuous advancements in technology, coupled with fundamental knowledge in chemistry, biology, and materials science.^1−3^ In 2012, on recognizing the considerable potential for nanotechnology to facilitate the development of sensitive, adaptable devices for detection, identification, and quantification of substances, the National Nanotechnology Initiative launched its fifth Nanotechnology Signature Initiative (NSI), entitled “Nanotechnology for Sensors and Sensors for Nano-technology: Improving and Protecting Health, Safety, and the Environment” (or the Sensors NSI).^4^ Engineered nanomaterials possess characteristics that might advance both the recognition and transduction steps of a probing event, as well as the signal-to-noise ratio, thanks to the miniaturization of the sensor elements.^5,6^ Thus, sensing at the nanoscale may be viewed as a natural fit. Nanomaterials with a high surface-to-volume ratio offer inherently high sensitivity to surface processes and lead to enhanced chemical reactivity, which can be modulated by the particle type, shape, and surface topography.^1^ Then, only a small number of analyte molecules are needed to produce a measurable signal, allowing both a reduction of sample volumes and a miniaturization of sensors.^7^ Moreover, the possibility to tailor nanomaterials with functional moieties confers precise sensitivity and specificity.^8^

Among other nanosensing platforms,^9−15^ gold nanoparticles (AuNPs) have inspired intensive efforts in the scientific community. Besides offering highly controllable sizes, shapes, and optical or electrical properties, they can be functionalized with a large variety of molecules involved in (bio)recognition with, for instance, oligonucleotides, antibodies, peptides, proteins, microorganisms, drugs, and other small molecules.^16−19^ In this regard, AuNPs capped with organic thiols are emerging as appealing chemical sensing tools.^20^ Thiolated ligands are known to bind strongly to gold surfaces and form self-assembled monolayers (SAMs). SAM-protected AuNPs (SAM-AuNPs) are thus stable multivalent systems, able to operate multiple molecular recognition events simultaneously at their surface.^21−27^

By changing ligands in the nanoparticle capping layer, it is possible to impart different chemical selectivities and sensitivities toward target analytes or groups of target analytes.^28−30^ Rotello and Bunz used cationic gold nanoparticles coated with different ammonium thiol derivatives to generate sensor arrays and polyanionic fluorescent polymers or proteins as indicators.^28^ This method was later expanded by Prins et al. to sense small polyanionic molecules.^29^ Based on a newly developed “NMR chemosensing” analytical approach, Mancin et al. demonstrated the ability of small gold nanoparticles passivated by a monolayer of amphiphilic thiols to detect salicylate molecules in a selective way. They could distinguish among a set of isomers, which differed only in the relative position of two functional groups, even when present in a mixture.^31^ By a combination of molecular dynamics (MD) calculations and magnetization-transfer NMR protocols, the authors proved the existence of transient binding pockets (for salicylate) in the monolayer with molecular features mimicking drug–protein recognition processes.^32,33^

Very recently, Gabrielli et al. have reported on a set of alkyl thiols bearing different terminal groups.^34^ If self-assembled on a ∼2 nm size gold core, they could detect and discriminate among a series of phenethylamine derivatives (designer drugs) in water, with estimated binding constants falling in the range of 1 × 10^5^–1.3 × 10^6^ M^–1^ for the most efficient system. An interesting point of this study is that it indicates the ability of rather nonspecific monolayers to discriminate chemically similar analytes, a sign of the complexity of noncovalent phenomena taking place on the monolayer.

To date, discovery of monolayers suitable for a specific sensing application is typically through trial and error, based on a handful of candidates, where ligand chemistry is the only criterion commonly adopted to modulate selectivity and sensitivity.^35^ Thus, deepening the knowledge of the basic principles governing molecular sensing at the monolayer surface has the potential to critically expand our ability to manipulate SAM-AuNP-based devices.

**Scheme 1:**
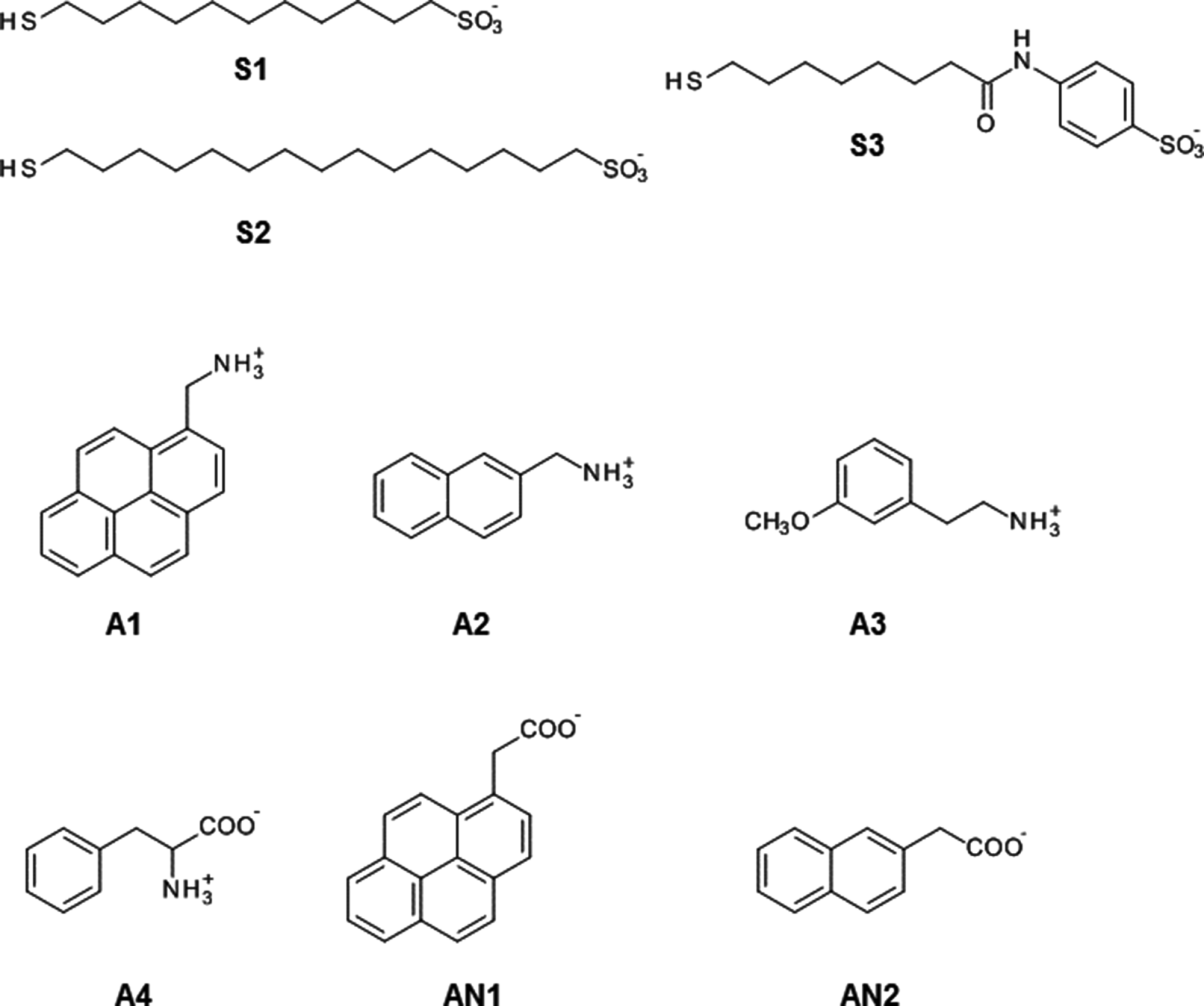
Structure of Nanoparticle-Coating Thiols (**S1**, **S2**, and **S3**) and Small Molecules Considered in This Work

To this purpose, taking advantage of the molecular view offered by MD calculations, we show how recognition occurs at the surface of three differently designed SAM-AuNPs, and we decipher which molecular features of both the coating ligand and monolayer affect the identification and discrimination of six small amphiphilic molecules (Scheme 1). Several of the thiols and compounds we consider here were previously tested experimentally by Gabrielli et al.^34^ for sensing phenethylamine derivatives (designer drugs) in water, and this also offers us the opportunity to dissect the influence of electrostatic and hydrophobic interactions, two major driving forces in supramolecular recognition. Moreover, for the first time, this study takes explicitly into account the role of monolayer organization and the solvent in mediating the interaction between SAM-AuNPs and small molecules. Even though the multivalent nature of these systems is an important feature and synergistic effects between binding sites could arise through cooperative recognition, we focus here on profiling molecular forces and ligand properties regulating the recognition of small molecules on SAM-AuNPs, leaving the detailed investigation of multivalency for a further study.

## Computational Methods

Ligands (**S1**, **S2**, and **S3**) and analytes (**A1**, **A2**, **A3**, **A4**, **AN1**, and **AN2**) were parametrized using antechamber, assigning gaff2 atom types,^36,37^ and their partial charges were derived by applying the RESP method provided by the RED server.^38^ The ligand protonation state was assigned based on a report of Gabrielli et al.^34^ Au–Au interactions were described with the parameters of the INTERFACE^39^ force field for metals. The Nanoparticle Builder module of OpenMD^40^ was used to generate an icosahedral gold cluster of 144 atoms, which models nanoparticles with an average core size of 1.6–1.8 nm.^41^ To preserve the geometry during simulation, all gold atoms within a distance of 2.90 Å were bonded to each other.^42^ Fifty^34^ sulfur headgroups and attached ligands were uniformly distributed on the gold surface;^43^ a harmonic bond was created between each sulfur atom and a gold atom within 3.3 Å.^42^ All Au–S and Au–Au bonds were modeled with a harmonic potential with a spring constant of 50.000 kJ/mol nm^2^.^42^ The interface structure disregards possible gold–sulfur binding motifs (e.g., staples, trimeric motifs, etc.); it has been shown recently^42^ that this simplified treatment yields a description of the structure of self-assembled alkanethiols of various lengths (*n* = 3–15) on a 2–6 nm size gold core in agreement with experiments.

Each solvated model (e.g., nanoparticles, analytes, and nanoparticle–analyte complexes) was prepared as described in the following paragraphs. Using the *tleap* program,^44^ the system was solvated with TIP3P water molecules, extending at least 20 Å from each solute atom; counterions were added to neutralize the system and match the experimental concentration.^34^ A combination of the steepest descent (10,000 cycles) and conjugate gradient methods (10,000 cycles) followed by a heating phase of 100 ps in the *NVT* ensemble (integration step = 1 fs) was carried out to reach the production temperature of 300 K. Then, density was brought to its final value with at least 50 ns in *NPT* conditions (integration step = 2 fs, pressure 1 atm), and pressure was maintained using a Berendsen barostat.^45^ Finally, we switched to the Monte Carlo barostat implemented in Amber for production run of which the first part was discarded until the steady state of the ligand RMSD was reached. The trajectory for final ensemble averages (400 ns) was stored from this point on. Temperature was controlled by the Langevin method (damping coefficient of 5 ps^–1^) throughout all simulations. Electrostatic interactions were computed by means of the Particle Mesh Ewald (PME)^46^ algorithm, and calculations were carried out using the AMBER 18^44,47^ suite of programs running on our hybrid CPU (minimization and heating) and GPU (all other steps) cluster^48,49^ (mixed precision). Each analyte–nanoparticle complex was built with 30 analyte molecules (as estimated experimentally^34^) placed randomly in the simulation box. Structural and energetic analysis was performed via AMBER programs pytraj, cpptraj, and MM-PBSA.py and by several in-house developed python scripts. Specifically, the SAM-AuNP/analyte free energy of binding Δ*G*_b_ was derived following the Molecular Mechanics/Poisson Boltzmann Surface Area (MM/PBSA) approach.^50^ It estimates the average interaction energy based on the solute molecular mechanics internal energy change (Δ*E*_MM_), solvation energy (Δ*G*_solv_), and conformational entropy change of the solute upon binding (−*T*Δ*S*). Δ*E*_MM_ consists of changes in the internal energies (Δ*E*_int_), electrostatic energy (Δ*E*_ele_), and van der Waals energy (Δ*E*_vdW_). The solvation energy term Δ*G*_solv_ includes two components: the electrostatic term (Δ*G*_p_solv_) and the nonpolar term (Δ*G*_np_solv_). The sum of Δ*E*_MM_ and Δ*G*_solv_ accounts for the enthalpy change associated with the binding (Δ*H*). Details on the calculation of each term are provided in the Supporting Information. The results were ensemble-averaged on three repeated and converged simulations.

## Results and Discussion

**Figure 1:**
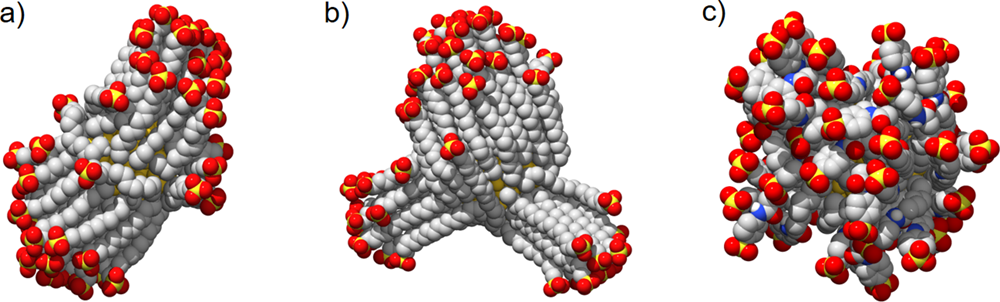
Monolayer organization as predicted by MD calculations for the three shells: (a) **S1-AuNP**, (b) **S2-AuNP**, and (c) **S3-AuNP**. Water molecules and ions are not displayed for clarity.

As a first step, we considered **S1**-functionalized AuNPs (**S1-AuNPs**) (Scheme 1). **S1-AuNPs** showed the highest affinities and remarkable selectivity in the experimental tests.^34^ MD simulations in water revealed that **S1** ligands self-organized around the gold core mainly into opposite oriented bundles (Figure 1), and only a limited amount of chains moved freely (see Table S1). Thus, the shell was elongated, and its shape was far from being spherical (see Table S1).

Spatially heterogeneous surfaces impact overall the NP behavior, especially their interfacial properties.^51−56^ We investigated the ability of **S1-AuNP** to bind and distinguish among three positively charged analytes (**A1**, **A2**, and **A3)** and one zwitterionic compound (**A4**) (see Scheme 1) having decreasing lipophilicity.

In addition, we considered compounds **AN1** and **AN2** (see Scheme 1). The negatively charged carboxylic group should make the interaction with the monolayer unfavorable, which could in turn be counterbalanced by the aromatic portion of the molecule. This wide spectrum range of analytes also allowed us to span the response of the computational approach in describing NP–small molecule recognition. In fact, despite the undoubted potential of these systems, to date, computational studies have been limited due to the complexity of sampling a multibinding event.^57^

**Figure 2:**
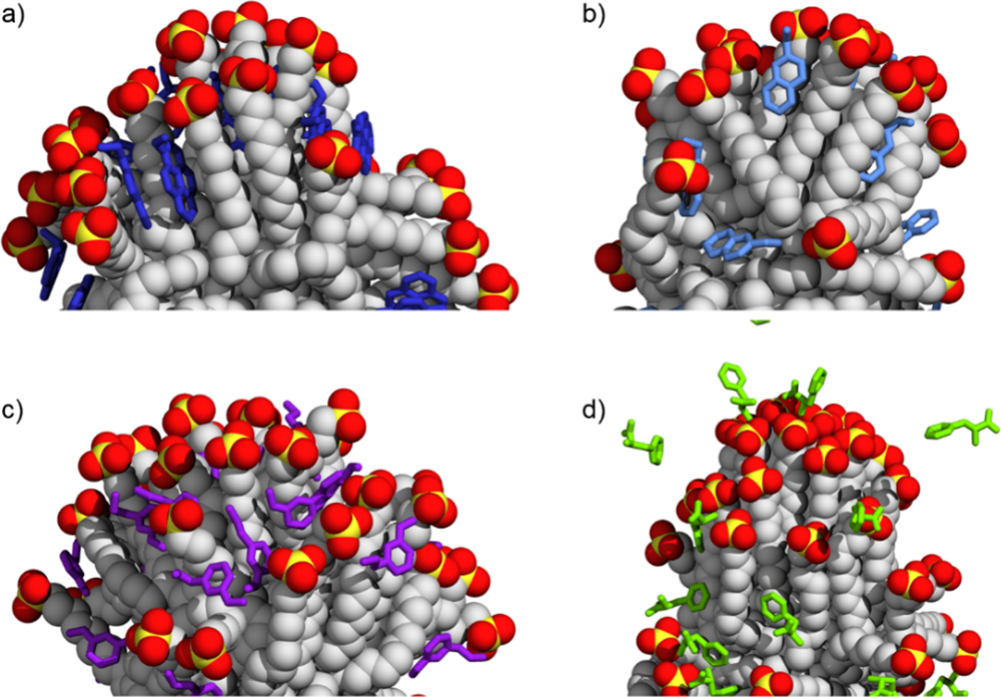
Selected configurations of **S1-AuNP** association with (a) **A1**, (b) **A2**, (c) **A3**, and (d) **A4** as obtained by MD calculations. Water and ions are not shown for the sake of clarity.

The simulations showed that **S1-AuNP** was able to associate effectively with the positively charged compounds (Figure 2). The contact was not permanent, but we observed binding and unbinding events. On average, all **A1** molecules interacted with **S1-AuNP**. The number of contacting molecules (i.e., at a distance lower than 0.5 nm from any heavy atom of the monolayer for times longer than 10 ns) decreased to 29 and 27 for **A2** and **A3**, respectively.

The three analytes associated with **S1-AuNP** in the same region of the monolayer (see Figure S1a).

Comparing the distribution of the sulfonate groups carried by the **S1** ligand with that of the amine groups **A1**–**A3**, we saw that they almost overlapped, suggesting a local interaction (see Figure S1b–d) that promotes the association. This likely arises from ion pairing and hydrogen bonding between SO_3_^–^ and NH_3_^+^ moieties as can be visually inferred from Figure S2. The complexation was also stabilized by the presence of a few water bridges between the aforementioned functional groups. The total number of salt and water bridges between **S1** and the three positively charged compounds was comparable in all systems (i.e., 26 on average), indicating that it is not specific to each analyte.

**Figure 3:**
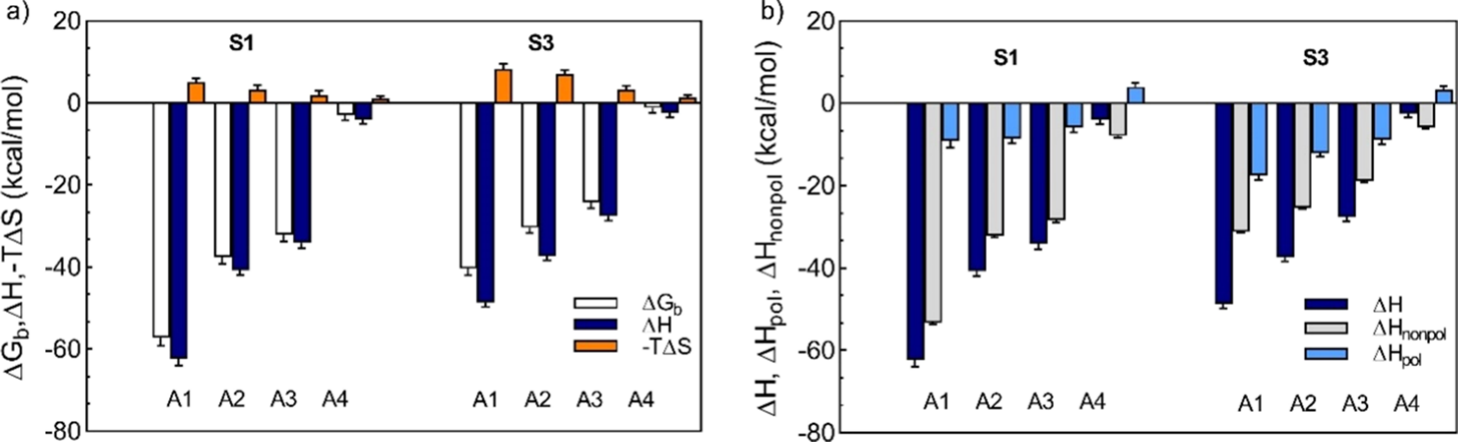
Thermodynamic binding signature of **S1-AuNP** and **S3-AuNP** to **A1**, **A2**, **A3**, and **A4**. (a) Binding free energy (Δ*G*_b_, white), enthalpy (Δ*H*, blue), and entropy (−*T*Δ*S*, orange) variation on an analyte basis. (b) Decomposition of total binding enthalpy (Δ*H*, blue) into nonpolar (Δ*H*_nonpol_, gray) and polar (Δ*H*_pol_, light blue) interaction changes. Δ*H*_pol_ accounts for electrostatics forces (Δ*E*_ele_) and polar contribution to solvation (Δ*G*_p_solv)_); Δ*H*_nonpol_ is the sum of van der Waals energy (Δ*E*_vdW_), nonpolar solvation (Δ*E*_np_solv_), and internal energy variation (Δ*E*_int_) terms.

Binding affinity is a straightforward measure of molecular recognition^58^ and can be computed by MD simulations. The MM/PBSA approach^50^ was used here to sample the bound states and evaluate the Gibbs binding energy Δ*G*_b_ of each analyte toward **S1-AuNP**. It is an end-point free energy method commonly used to compute the binding free energies of small molecules to large biomolecule receptors as well as to describe large interbiomolecular recognitions.^59−64^ Moreover, it allows to decompose Δ*G*_b_ in its enthalpic (Δ*H*) and entropic (−*T*Δ*S*) terms and to reveal the molecular forces that drive the binding. This thermodynamic signature is reported in Figure 3a.

For **A1**–**A4** compounds, the enthalpy change was favorable (Δ*H* < 0), whereas entropy variation opposed binding (−*T*Δ*S* > 0). However, the entropic penalty paid was outweighed by the enthalpy gain, thus suggesting that enthalpy is the major driving force for complex formation. This is also a hallmark of the so-called enthalpy–entropy compensation mechanism observed widely in (bio)molecular complexes.^65,66^

Pleasingly, the predicted affinity trend agrees well with the experimental counterpart^34^ (see also Table S2 and Figure S3), with **A1** outperforming **A2** and **A3** also in the calculations.

Polar contribution (Δ*H*_pol_) to enthalpy, which arises from Coulombic interactions between **S1-AuNP** and each analyte and polar solvation energy, was always favorable for binding (see Figure 3b). Thus, the unfavorable desolvation of polar groups was compensated for by favorable intermolecular electrostatic interactions. For all positively charged analytes, Δ*H*_pol_ yielded contribution in the range 4–9 kcal/mol and accounted for only a small fraction of the total binding enthalpy. Strong intermolecular van der Waals interactions and hydrophobic forces are instead required for boosting the molecular recognition. Indeed, Δ*H*_nonpol_ was the dominant energetic contribution in association and the main interaction responsible for the observed selectivity of **S1-AuNP**.

The complementarity of electrostatic and hydrophobic interactions in driving complex formation on SAMs is even more evident, including the binding of the zwitterionic (**A4**) and negatively charged compounds (**AN2**, **AN1)** in the discussion. **A4** is close to **A3** in terms of log *D* values (−1.46 and −1.04, respectively) but bears a negatively charged carboxylic group besides a positively charged amine. This had a dramatic effect on the affinity and led to a decrease (less negative value) of Δ*G*_b_ from −32.2 ± 1.6 kcal/mol for **A3** to −3.0 ± 1.2 kcal/mol for **A4**. The reduced affinity of **A4** was also seen by Gabrielli et al. in the NOE pumping spectra, where **A4** did not produce any signal.^34^ The polar contribution (Δ*H*_pol_) became positive (unfavorable); at the same time, the nonpolar term (Δ*H*_nonpol_) reduced significantly, overall accounting for a much less effective enthalpic stabilization. As a consequence, the number of **A4** molecules temporarily making contact with **S1-AuNP** dropped down to 19.

At the same time, none of the negatively charged analytes (**AN1** and **AN2)** bound significantly to **S1-AuNP**. Both transiently approached the nanoparticle on the surface (see Figure S4), but their free energy of binding was positive (Δ*G*_b_ = 1.40 ± 0.8 kcal/mol for **AN1** and 5.3 ± 1.2 kcal/mol for **AN2**), evidencing that the association with anionic amphiphilic molecules is not favored by thermodynamics.

Taken together, these findings provide clues that electrostatic interactions between oppositely charged species are needed to drive analytes toward their optimal binding mode; hydrophobic forces that originate from the interaction of aromatic units in the hydrophobic portion of the shell stabilize the complex and modulate the affinity of **S1-AuNP** toward the binding partner. A precise combination of these two forces thus appears as a way to control the overall affinity and specificity.

Among positively charged analytes, the binding mode of **A1** deserves a specific discussion. At a closer look, while some molecules interacted with the monolayer at the water–bundle interface, the majority of **A1** resided inside the bundles with the aromatic rings oriented parallel to the hydrophobic backbone of **S1** (see Figure S5a). This specific placement allows optimization of the van der Waals forces and may explain the highest affinity (Δ*G*_b_) of this compound due to a more favorable nonpolar contribution (Δ*H*_nonpolar_) to association (see Figure 3a). For comparison, **A2**, which features only a naphthalene moiety, is not able to create a pattern of hydrophobic interactions with the extent similar to that observed for **A1** (see Figure S5b). To test the efficiency of this recognition mechanism, we then considered **S2-AuNP** (see Scheme 1). **S2-AuNP** has the same core size of **S1-AuNP**, but the alkyl chain in **S2** is longer than that in **S1** (16 vs 11 carbon atoms, respectively). This endows ligands with a higher degree of freedom and the nanosensor with greater hydrophobic potential. **S2-AuNP** presented a monolayer organized in bundles (see Figure 1b) with a few free chains (see Table S1). Binding of **A1** altered the monolayer organization: due to their inherent flexibility and small NP core size, the chains fluctuate and bend over the gold core to optimize ligand–ligand and ligand–**A1** multiple interactions (see Figure S6). This reduced the number of bundles in the monolayer upon binding (see Table S3). Still, **A1** resided among ligands as seen for **S1-AuNP**, thus confirming the strength and role of ligand–analyte parallel pairing in leading the molecular interaction.

The behavior of **S2-AuNP** also raises another important issue commonly neglected when designing new ligands for supramolecular sensors based on SAM-AuNPs: the role of ligand flexibility and collective/individual loss (or gain) in entropy upon the recognition and the consequent negative (or positive) contribution to the total free energy of binding. The computed entropy change (−*T*Δ*S*) for all **S1-AuNP** complexes (see Figure 3a) was positive, indicating a loss of conformational flexibility of the binding partners, which is unfavorable for binding. This cost was lower for **A3** and **A4** than for **A1** and **A2** complexes. **A3**/**A4** compounds had a lower affinity toward **S1-AuNP**, and **A4** also showed a decreased number of bound molecules, overall resulting in a smaller effect on chain mobility. Consistently, the structural features of **S1-AuNP** were closer to those of the unbound nanoparticle for **S1-AuNP/A3** and **S1-AuNP/A4** systems (see Table S4).

This first set of calculations provides us with unprecedented molecular details into factors affecting the ability of a self-assembled monolayer to discriminate between small target molecules on spherical surfaces. Yet, molecular recognition is a two-player game, and acquiring a complete picture is possible only by exploring the binding also from the nanoparticle perspective, i.e., changing its covering ligands. Fluorescence titration experiments performed by Gabrielli et al.^34^ assessed that modification of the coating thiol with an aromatic head group (**S3**, see Scheme 1) results in a reduction of the affinity for each analyte, compared to **S1-AuNP** (see Table S2). However, no comprehensive molecular rationale was attempted at that time.

**Figure 4:**
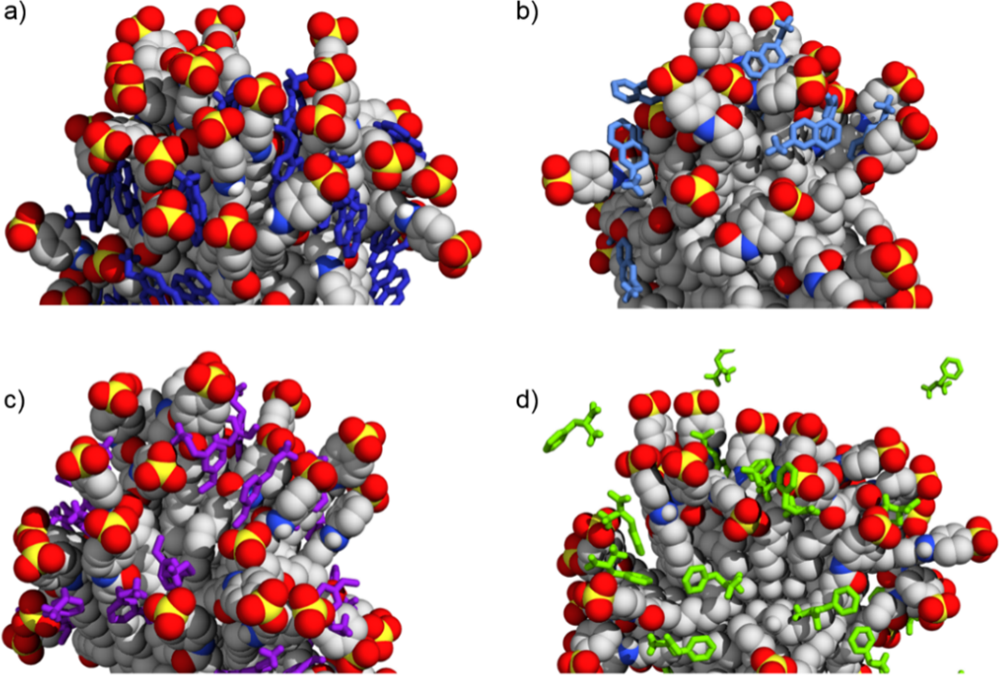
Selected configurations of **S3-AuNP** association with (a) **A1**, (b) **A2**, (c) **A3**, and (d) **A4** as obtained from MD calculations. Water and ions are not shown for the sake of clarity.

Our MD calculations predicted an essentially spherical organization of **S3** ligands around the gold core (see Figure 1c and Table S1) devoid of any chain bundling (contrary to **S1** and **S2** ligands), which can be ascribed to the presence of a bulkier headgroup and shorter length of the alkyl chain that restrict ligand association.^67^ Considering again the three positively charged (**A1**, **A2**, and **A3**) and zwitterionic (**A4**) compounds (see Figure 4), the average number of molecules bound was comparable to that found for **S1-AuNP** as well as the total number of ion pairs and water bridges among **S3** chains and each analyte (see Table S5), suggesting that these features are only modestly influenced by the ligand chemistry in the systems investigated here.

The binding thermodynamics (see Figure 3) indicated that the entropic term still caused an energetic penalty; its value was larger than that found for the corresponding **S1-AuNP** systems. In the unbound state, the absence of bundles endowed thiols with a higher degree of mobility and flexibility; when analytes approach the monolayer, they hinder ligand natural conformational fluctuations more than those in **S1-AuNP**, leading to an increased entropic cost. Reasonably, this decreased with the affinity toward each analyte. At the same time, we observed a significant reduction in the enthalpy contribution to binding. Coupling these two effects led to a reduced affinity of **S3-AuNP** toward each compound if compared to **S1-AuNP**, which matches the experimental findings^32^ (see also Table S2 and Figure S3). A summary of the structural characterization of **S3-AuNP** upon **A1**, **A2**, **A3**, and **A4** binding can be found in Table S6 and Figure S7. Again, none of the negatively charged analytes (**AN2** and **AN1)** was detected proficiently by **S3-AuNP**, showing positive Δ*G*_b_ values of 4.1 ± 1.0 and 1.1 ± 0.5 kcal/mol, respectively, highlighting the contribution of electrostatic interactions in recognition processes involving SAMs and small charged molecules.

**Figure 5:**
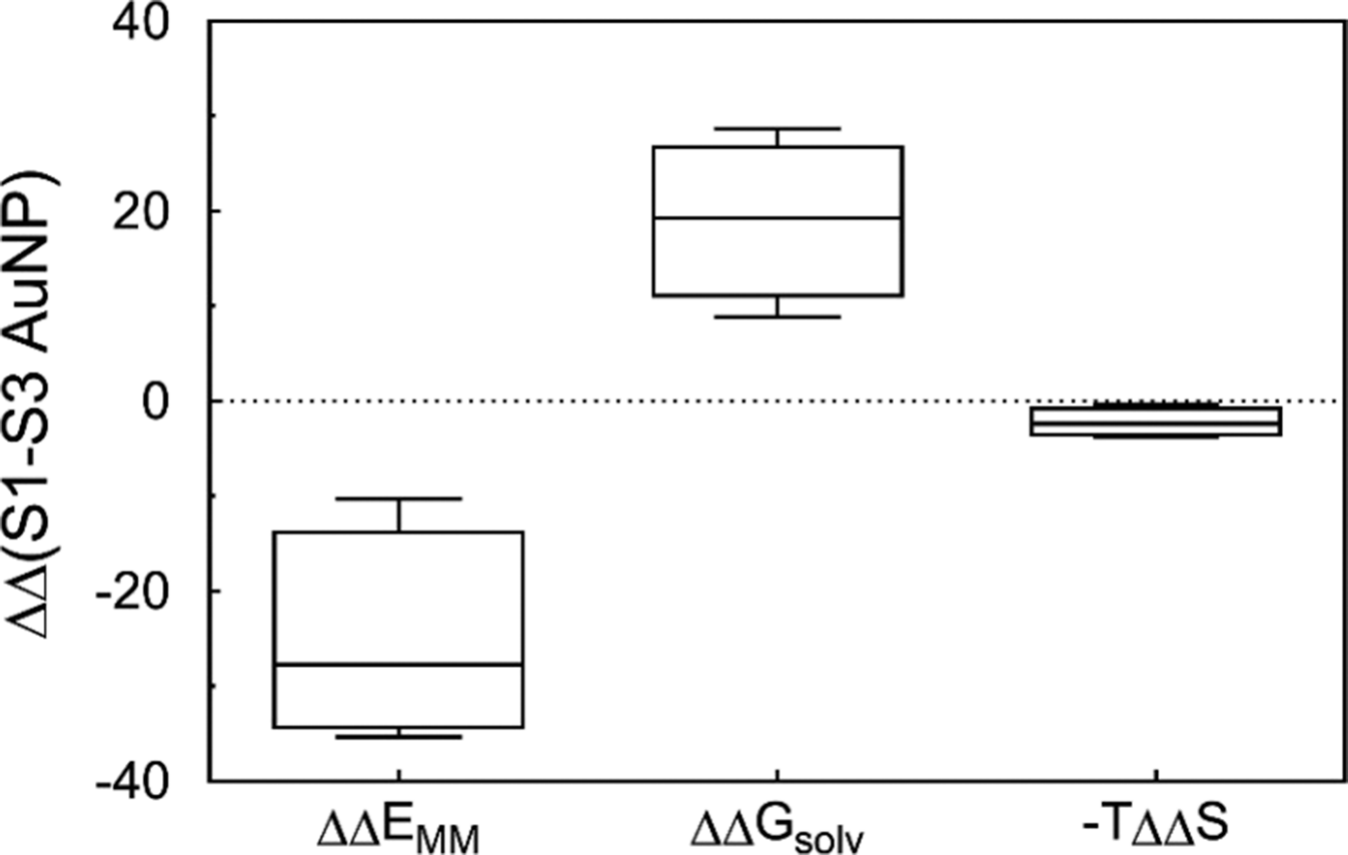
Box plot of the difference in terms of Δ*EG*_MM_, Δ*G*_solv_, and −*T*Δ*S* between **S1-AuNP** and **S3-AuNP** once bound to **A1**, **A2**, **A3**, and **A4**.

In contrast to **S1-AuNP**, both polar and nonpolar terms contributed to ΔH, and we sought where these differences may arise. Figure 5 shows the difference in terms of Δ*E*_MM_, Δ*G*_solv_, and −*T*Δ*S* between **S1**-**AuNP** and **S3-AuNP** bound to **A1**, **A2**, **A3**, and **A4**. Although the change in entropy was obviously dissimilar between the two systems, it did not represent the major contribution to recognition. Intermolecular (and intramolecular) interactions (Δ*E*_MM_) were mainly responsible for the differing affinity of these systems toward the same analyte. Clearly, the chemical structure of the coating ligands determines the ability to establish more (or less) favorable interactions with a binding partner. At the same time, solvent-mediated forces (Δ*G*_solv_) were equally responsible for the different interaction abilities.

**Figure 6:**
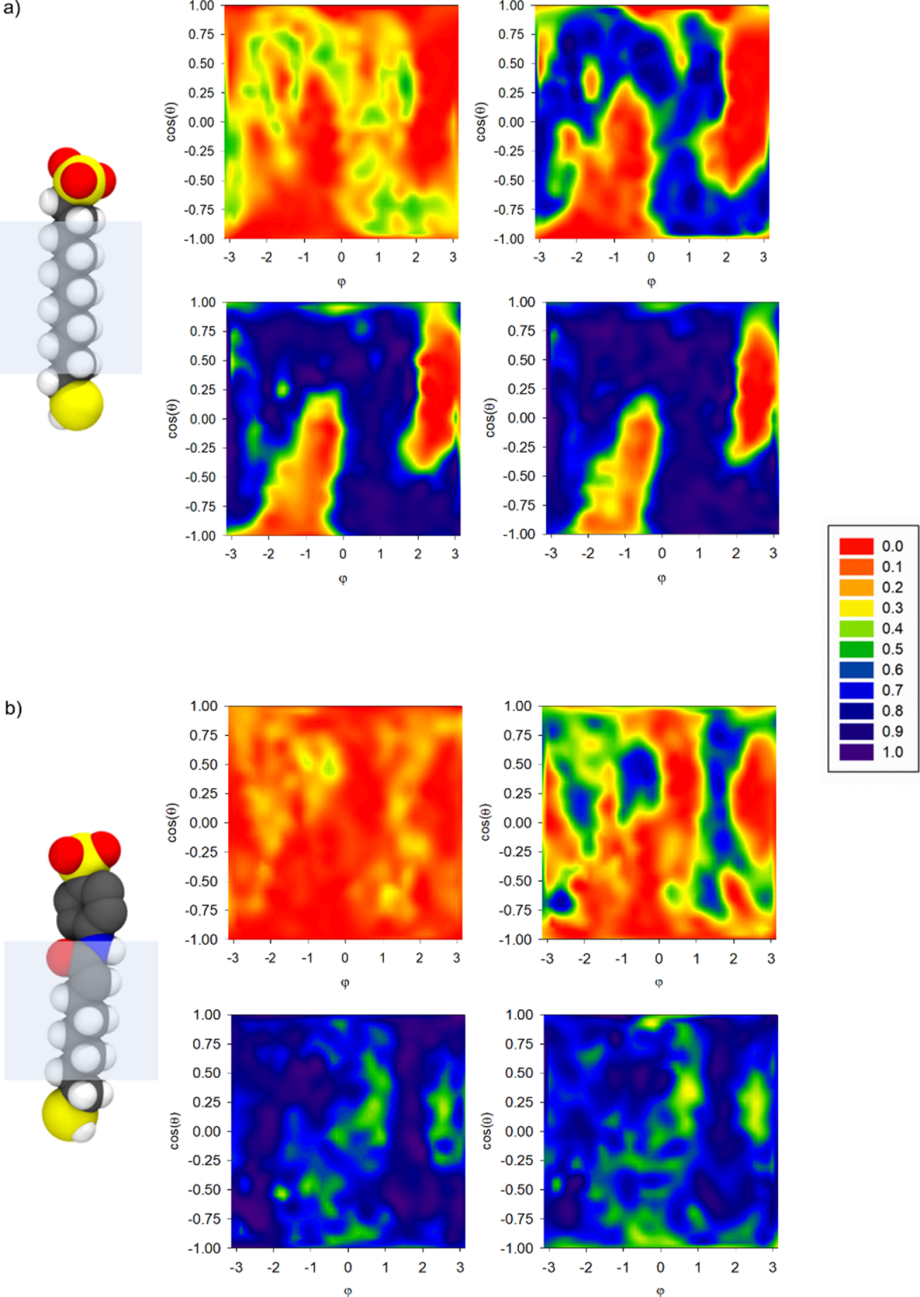
Normalized water distribution at increasing distance from the gold surface (∼4 to 10 Å up-left to right-down panels) for (a) **S1-AuNP** and (b) **S3-AuNP**. The graphs plot the distribution of the atom (oxygen of water or carbon of thiols) closest to spherical surfaces (centered on the gold core and placed at increasing distances from the NP core) shown as a two-dimensional projection of the sphere surface (*x* axis, the azimuthal angle φ; *y* axis, the cosine of the polar angle θ). A value of 1 indicates that an oxygen atom of a water molecule is always the closest; if it is equal to 0, it indicates that a carbon atom of a chain is always the closest.

Figure 6 shows the comparison of averaged density distributions of water around the core for **S1**-**AuNP** and **S3-AuNP** at several distances from the gold surface. It is evident that different monolayer morphologies led to dissimilar nanoparticle hydration within the monolayer. For **S1-AuNP,** the aggregation of ligands made the distribution of water molecules around the nanoparticle spatially heterogeneous, with areas less hydrated or not accessible to the solvent, for instance, inside the bundles (red areas in Figure 6a).

Vice versa, when the organization of the monolayer is disordered (as in **S3-AuNP**, see Figure 6b), the penetration of the solvent within the shell was higher and more uniform. Thus, the presence of bundles induces a hydrophobic environment that favors the recognition of small amphiphilic molecules.

This observation is consistent with recent evidence by van Lehn et al. on planar SAMs.^68−70^ The authors proved that spatially disordered SAMs affect the interfacial properties of the water solvent and decrease the interfacial hydrophobicity with respect to ordered surfaces. Such a phenomenon not only explains the marked positive difference in the solvation term Δ*G*_solv_ between **S1**-**AuNP** and **S3-AuNP** (see Figure 5) but also brings out the active contribution of the solvent in the recognition mechanism between SAMs and small amphiphilic molecules on curved surfaces.

Summing all up, the reduced affinity of **S3-AuNP** with respect to **S1**-**AuNP** stems from several concomitant factors: first, the different ligand chemistry responsible for less effective interactions as evidenced by ΔΔ*E*_MM_; second, the dissimilar ligand flexibility, which modulates the binding affinity toward an analyte through different entropy costs, higher for **S3-AuNPs**; and last, the different monolayer organization—the disordered shell in **S3-AuNP** offers a less hydrophobic solvation microenvironment, which disfavors the partition of amphiphilic analytes.

## Conclusions

Sensing platforms based on self-assembled monolayers (SAMs) of organic thiols on gold nanoparticles are multivalent and cooperative systems whose strength and selectivity toward selected substrates can be tailored by designing ad hoc the monolayer constituents. To that end, mastering the basic principles that regulate recognition at the monolayer surface is needed. In this paper, we have investigated three different SAMs and by means of molecular dynamics calculations have analyzed their ability to detect and discriminate a set of small amphiphilic charged molecules from a molecular perspective. The chosen SAMs are deprived of any structural and chemical feature that would permit specific interactions, allowing us to explore the underlying forces and molecular attributes that shape the formation of such supramolecular complexes.

Our comprehensive investigation reveals that probing small molecules with SAMs on curved surfaces is a complex, multidimensional phenomenon distinct from that occurring on planar SAMs. It is regulated by either single-molecule properties (such as ligand chemistry and flexibility) or collective features (such as SAM organization and presence of interfaces). Moreover, the same binding event may significantly alter the monolayer structure, thus adding another level of complexity. We also showcase that the shell structure influences the solvation interfacial microenvironment through combined hydrophobic interactions, which may be tuned to tailor the affinity.

We believe that the acquired knowledge of the intimate mechanisms driving sensing at the SAM surface will expand our ability to manipulate and computationally design nanodevices with enhanced recognition ability toward small molecules, such as drugs, metabolites, or small molecular markers for cancer.

## Supporting Information Available

Details on MM-PB/SA methodology and additional results (PDF)
