# Supplementary material for: Molecular Features for Probing Small Amphiphilic Molecules with Self-Assembled Monolayer-Protected Nanoparticles
Source: Langmuir. 2020 Apr 29;36(20):5671–9. doi: 10.1021/acs.langmuir.9b03686 (PMC8007095; doi:10.1021/acs.langmuir.9b03686)
Supplement: Supplementary file 1 — la9b03686_si_001.pdf [file la9b03686_si_001.pdf]

Molecular Features for Probing Small Amphiphilic Molecules with Self-Assembled Monolayer Protected Nanoparticles

Domenico Marson,[a] Zbyšek Posel,[a,b] and Paola Posocco[a*]

[a] *Department of Engineering and Architecture, University of Trieste, Italy*

[b] *Department of Informatics, Faculty of Science, Jan Evangelista Purkyně University, Ústí nad Labem, Czech Republic*

*paola.posocco@dia.units.it

INDEX

Additional results 2

Computational details 7

ADDITIONAL RESULTS

Table S1. Summary of the structural characterization in solvent for the three SAM-AuNPs considered in this study, including: nanoparticle radius of gyration (R_g); average number of ligand bundles; number of free chains; asphericity (δ) and relative ratio of the principal moments of inertia (I_z/I_x, I_z/I_y); fraction of *trans* dihedrals angles. Uncertainties are reported in brackets.

	R_g (nm)	Average number of bundles[a]	Free chains[b]	δ[c] (-)	I_z/I_x[c] (-)	I_z/I_y[c] (-)	Fraction of *trans* dihedrals (%)[d]
**S1-AuNP**	1.50 (0.01)	2	9.0 (4.8)	0.86 (0.13)	3.25 (0.35)	2.34 (0.40)	89.3 (2.7)
**S2-AuNP**	1.77 (0.02)	3	2.7 (1.4)	0.73 (0.09)	3.74 (0.30)	1.29 (0.13)	93.0 (3.4)
**S3-AuNP**	1.54 (0.01)	no bundles	47.1 (2.6)	0.32 (0.07)	1.68 (0.17)	1.30 (0.12)	79.8 (5.6)

[a,b] Ligands are assigned to the same bundle based on their relative orientation and end group distances; we used the Hierarchical Density-based Spatial Clustering of Applications with Noise (HBDSCAN)[1] algorithm to identify sets of ligand that belong to the same bundle. We assigned a minimum number of 5 ligands to form a bundle. Chains not pertaining to a specific bundle were considered as “free”. Low deviation from average values suggests that the bundling structure is rather stable on the time scale inspected (400 ns). Monolayer structures formed consistently in each system replicate.

[c] The asphericity δ gives an indication of shape and is defined as I_z-(I_x+I_y)/2, having defined the principal moments of the gyration tensor as $I_z \geq I_y \geq I_x$. Values close to 0 indicate a spherical form, while values around 1 an oblong shape (e.g., ellipsoid).

[d] The fraction of *trans* dihedrals angles (-180° < φ < -120° and 120° < φ < 180°) relative to the total number of dihedral angles in the ligand chain is a measure of ligand ordering. The dihedral angles were calculated taking into account all the heavy atoms of the alkyl portion and ignoring all the hydrogen atoms. A fraction of *trans* dihedral angles values near 1 indicates a highly ordered ligand structure and a value near 0 a highly disordered structure.

Presence of bundles is already documented for other alkanethiols either experimentally and computationally and is related to the number of ligand carbon atoms, terminal moiety, and gold core size.[2-9]

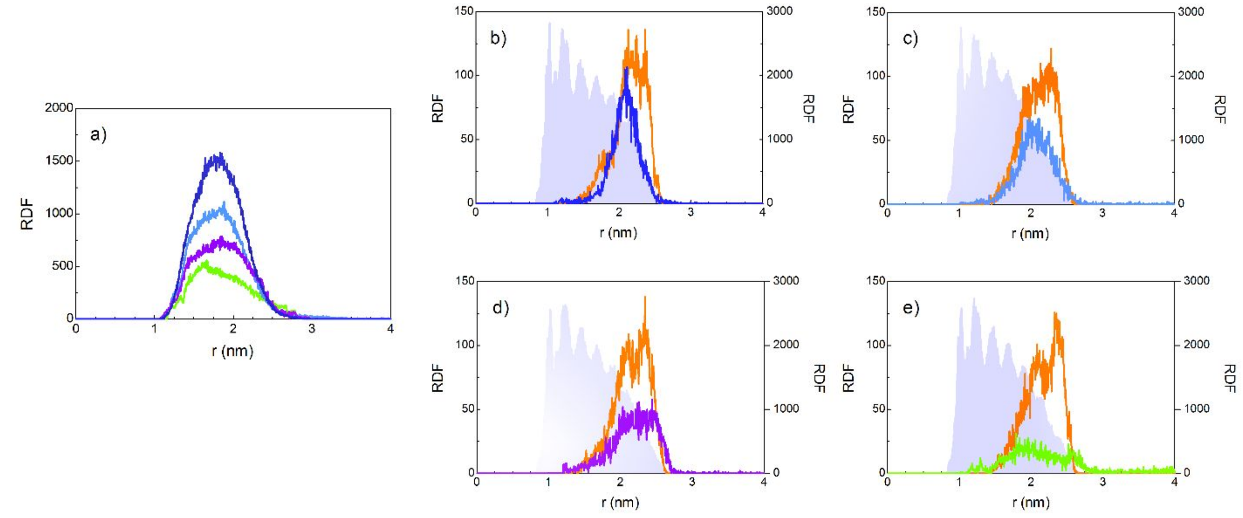

Figure S1. Distribution of A1, A2, A3 and A4 around the gold core for S1-AuNP based systems. (a) Radial distribution function (RDF) of **A1** (blue), **A2** (light blue), **A3** (purple) and **A4** (chartreuse) calculated from the center of mass of the nanoparticle. (b-e) RDF of sulfonate moieties in **S1** ligands (orange) and amine groups in **A1** (blue), **A2** (light blue), **A3** (purple) and **A4** (chartreuse) calculated from the center of mass of the nanoparticle (left axis). For comparison the RDF of the whole **S1** ligand is also reported as a grey area (right axis).

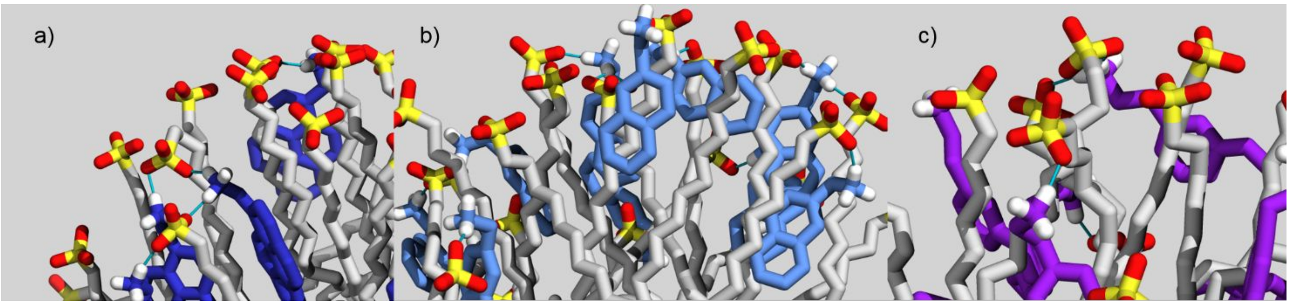

Figure S2. Selected examples of salt-bridging in **A1** (a), **A2** (b), and **A3** (c) bound to **S1-AuNP**. **S1** ligands are depicted as stick and colored by element. **A1**, **A2**, and **A3** are represented in blue, light blue, and purple sticks, respectively. Salt-bridges are highlighted in cyan.

Table S2. Binding free energy (ΔG_b)(kcal/mol) and components calculated with MM/PBSA approach and binding constant (K)(M^{-1}) taken from Gabrielli et al.[10] of analytes **A1**, **A2**, **A3**, **A4** to **S1-AuNP** and **S3-AuNP** at room temperature.

System	ΔH_{nonpol}	ΔH_{pol}	ΔH	$-T\Delta S$	ΔG_b	K
**S1-AuNP/A1**	-53.2±0.3	-9.1±1.6	-62.3±1.6	5.0±1.0	-57.3±1.9	$(2.2\pm 0.1)\times10^6$
**S1-AuNP/A2**	-32.1±0.5	-8.6±1.1	-40.7±1.2	3.2±1.1	-37.5±1.6	$(6.1\pm1.4)\times10^5$
**S1-AuNP/A3**	-28.4±0.4	-5.8±1.2	-34.2±1.3	1.9±1.0	-32.3±1.6	$(4.1\pm1.2)\times10^5$
**S1-AuNP/A4**	-7.9±0.4	4.0±0.9	-3.9±1.04	0.9±0.6	-3.0 ±1.2	no binding observed
**S3-AuNP/A1**	-31.2±0.2	-17.5±1.1	-48.7±1.1	8.3±1.2	-40.4±1.6	$(1.3\pm0.1)\times10^6$
**S3-AuNP/A2**	-25.3±0.2	-12.1±0.8	-37.4±0.9	7.0±1.0	-30.4±1.3	$(5.1\pm0.3)\times10^5$
**S3-AuNP/A3**	-18.9±0.3	-8.8 ±1.1	-27.7±1.1	3.3±0.9	-24.4 ±1.4	$(9.3\pm1.2)\times10^4$
**S3-AuNP/A4**	-5.8±0.3	3.3±0.9	-2.5±1.0	1.3±0.6	-1.2±1.1	-

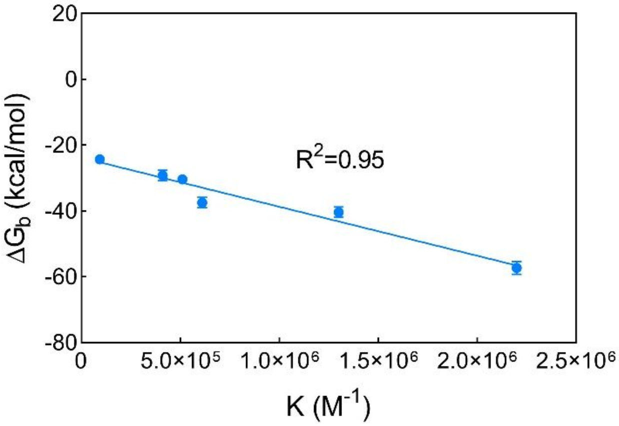

Figure S3. Correlation between free energy of binding (ΔG_b) and affinity constant (K) for systems in Table S2.

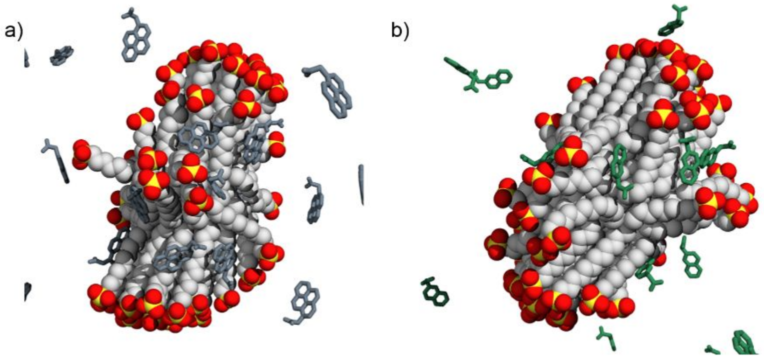

Figure S4. MD snapshots of AN1 (a) and **AN2** (b) with **S1-AuNP**. **S1** ligands are depicted as spheres and colored by element. **AN1** and A**N2** are represented in grey and green sticks, respectively. Water and ions are not shown for the sake of clarity.

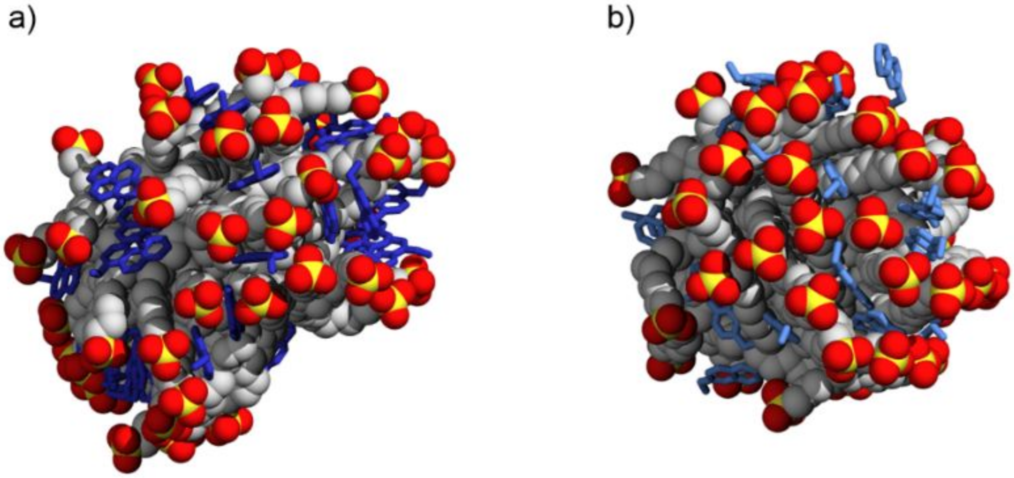

Figure S5. Top view showing interligand placement of A1 (a) and A2 (b) when bound to S1-AuNP. S1 ligands are depicted as spheres and colored by element. **A1** and **A2** are represented in blue and light blue sticks, respectively. Water and ions are not shown for the sake of clarity.

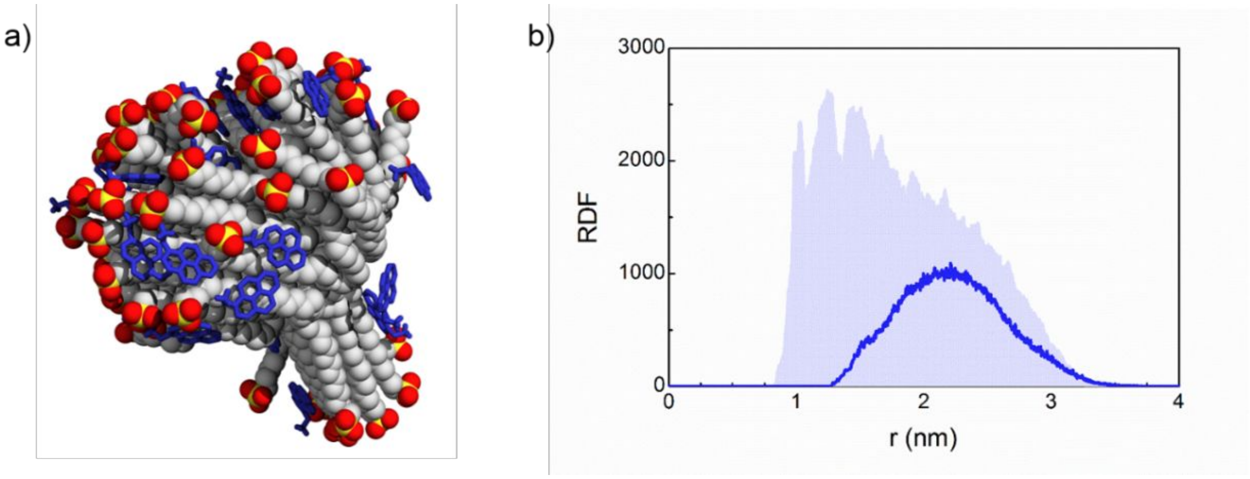

Figure S6. (a) MD snapshots of A1 with **S2-AuNP**. **S2** ligands are depicted as spheres and colored by element. **A1** is represented in blue sticks. Water and ions are not shown for the sake of clarity. (b) Radial distribution function (RDF) of **S3** ligands (grey) and **A1** (blue) calculated from the center of mass of the nanoparticle.

Table S3. Structural characterization of S2-AuNP upon binding of A1, including: nanoparticle radius of gyration (R_g); average number of ligand bundles; number of free chains; asphericity (δ) and relative ratio of the principal moments of inertia (I_z/I_x, I_z/I_y); fraction of *trans* dihedrals angles. Uncertainties are reported in brackets.

	R_g (nm)	Average number of bundles	Free chains	δ (-)	I_z/I_x (-)	I_z/I_y (-)	Fraction of *trans* dihedrals (%)
**A1**	1.78 (0.01)	2	1.8 (1.1)	0.81 (0.11)	3.61 (0.26)	1.43 (0.17)	91.9 (3.3)

Table S4. Structural characterization of S1-AuNP upon A1, A2, A3 and A4 binding, including: nanoparticle radius of gyration (R_g); average number of ligand bundles; number of free chains; asphericity (δ) and relative ratio of the principal moments of inertia (I_z/I_x, I_z/I_y); fraction of *trans* dihedrals angles. Uncertainties are reported in brackets.

	R_g (nm)	Average number of bundles	Free chains	δ (-)	I_z/I_x (-)	I_z/I_y (-)	Fraction of *trans* dihedrals (%)
**A1**	1.56 (0.01)	2	4.4 (2.9)	0.58 (0.12)	2.31 (0.22)	1.70 (0.29)	88.8 (2.7)
**A2**	1.57 (0.01)	2	3.3 (2.3)	0.65 (0.12)	2.48 (0.26)	1.88 (0.29)	89.2 (2.5)
**A3**	1.55 (0.01)	2	9.9 (8.2)	0.90 (0.09)	3.08 (0.26)	2.51 (0.27)	85.6 (2.7)
**A4**	1.54 (0.02)	2	9.8 (5.3)	0.82 (0.09)	3.11 (0.31)	2.13 (0.26)	89.7 (2.6)

Table S5. Number of contact molecules (N_c) and total number of ion pairs and water bridges (N_h) for **S1** and **S3-AuNP** in complex with **A1**, **A2**, **A3**, and **A4**.

	**A1**		**A2**		**A3**		**A4**	
	N_c	N_h	N_c	N_h	N_c	N_h	N_c	N_h
**S1-AuNP**	30 ± 0	27 ± 4	29 ± 1	26 ± 5	27 ± 2	26 ± 4	19 ± 3	16 ± 4
**S3-AuNP**	30 ± 0	27 ± 4	29 ± 1	28 ± 2	29 ± 1	27 ± 3	17 ± 3	21 ± 5

Table S6. Structural characterization of S3-AuNPs upon A1, A2, A3 and A4 binding, including: nanoparticle radius of gyration (*R_g*); average number of ligand bundles; number of free chains; asphericity (*δ*) and relative ratio of the principal moments of inertia (I_z/I_x, I_z/I_y); fraction of *trans* dihedrals angles. Uncertainties are reported in brackets.

	R_g (nm)	Average number of bundles	Free chains	δ (-)	I_z/I_x (-)	I_z/I_y (-)	Fraction of *trans* dihedrals (%)
**A1**	1.56 (0.01)	no bundles	45.2 (2.7)	0.33 (0.06)	1.64 (0.11)	1.35 (0.10)	81.3 (7.7)
**A2**	1.56 (0.02)	no bundles	48.1 (1.9)	0.31 (0.07)	1.69 (0.17)	1.25 (0.12)	79.7 (8.7)
**A3**	1.54 (0.01)	no bundles	46.4 (2.5)	0.27 (0.07)	1.57 (0.15)	1.24 (0.12)	81.0 (6.3)
**A4**	1.56 (0.02)	no bundles	47.0 (2.2)	0.35 (0.10)	1.89 (0.29)	1.25 (0.15)	80.3 (6.9)

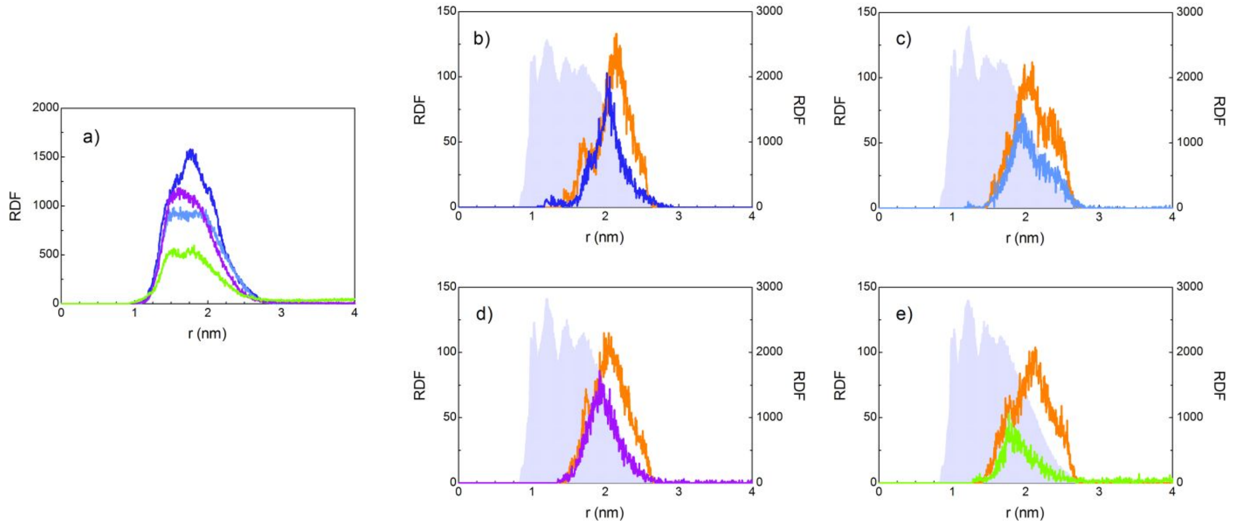

Figure S7. Distribution of A1, A2, A3 and A4 around the gold core for S3-AuNP based systems. (a) Radial distribution function (RDF) of **A1** (blue), **A2** (light blue), **A3** (purple) and **A4** (chartreuse) calculated from the center of mass of the nanoparticle. (b-e) RDF of sulfonate moieties in **S3** ligands (orange) and amine groups in **A1** (blue), **A2** (light blue), **A3** (purple) and **A4** (chartreuse) calculated from the center of mass of the nanoparticle (left axis). For comparison the RDF of the whole **S3** ligand is also reported as a grey area (right axis).

COMPUTATIONAL DETAILS

Molecular mechanics Poisson–Boltzmann surface area (MM/PBSA) methodology

Binding free energies were computed applying the MM/PBSA methodology. Details on the methodology were reviewed recently in [11,12] and are summarized here in the following paragraphs. According to the MM/PBSA approach, the free energy of binding an analyte (A) to a nanoparticle (N) to form a complex (NA) in solution ($\Delta G_{b,solv} = \Delta G_b$)

$$\Delta G_{b,solv} = \Delta G_{NA,solv} - (\Delta G_{N,solv} + \Delta G_{A,solv}) \quad (1)$$

can be expressed as

$$\Delta G_{b,solv} = \Delta H - T\Delta S = \langle \Delta E_{MM} \rangle + \langle \Delta G_{sol} \rangle - T\langle \Delta S_{solute} \rangle \quad (2)$$

ΔE_{MM} represents the molecular mechanical energy change in gas phase, ΔG_{solv} the solvation free energy change, and -TΔS_{solute} (= -TΔS) the conformational entropy change of the solute upon binding.

ΔE_{MM} includes three terms:

$$\Delta E_{MM} = \Delta E_{int} + \Delta E_{ele} + \Delta E_{vdW} \quad (3)$$

the changes in the internal energies ΔE_{int} (bond, angle, and dihedral energies), the electrostatic energy change ΔE_{ele}, and the van der Waals energy change ΔE_{vdW}.

ΔG_{solv} is separated into electrostatic solvation energy ΔG_{p_solv} (polar contribution) and nonpolar contribution ΔG_{np_solv} between the solute and the continuum solvent. The polar contribution ΔG_{p_solv} was calculated using an implicit solvent model, here the Poisson−Boltzmann (PB) model[12]. The nonpolar solvation energy arises from the solute cavity formation within the solvent and van der Waals interactions between the solute and the solvent around the cavity and is estimated using a fast LCPO algorithm.[13] The surface tension (γ) and correction term (b) were set to 0.00542 kcal*mol^{-1}*Å^{-2} and 0.92 kcal*mol^{-1} values, respectively. The change in conformational entropy $-T\Delta S_{solute}$ was computed by normal-mode analysis as implemented in MM-PBSA.py and *nmode* programs of AmberTools18. For free energy calculation, a solute dielectric constant ε_{in} = 2 was employed to account for the hydrophobic nature of the inner portion of the monolayer.

Angle brackets in equation (2) indicate averages from an ensemble of representative structures, obtained extracting relevant frames from an equilibrated molecular dynamics trajectory. The

computation for the non-entropic terms was averaged over 100 configurations evenly extracted from data collection trajectories, while the entropic term was averaged over a subset of 10 configurations (out of 100) for each independent simulation.Three replicates were considered for each system.

To account for binding-induced conformational changes, we applied a “multi-trajectory”[14] approach taking three trajectories from three independent simulations (the nanoparticle in solution, the analytes in solution, and the nanoparticle-analytes complex in solution) to compute the different terms in equation (1).

REFERENCES

1. (a) Campello, R. J. G. B.; Moulavi, D.; Sander, J. In *Density-Based Clustering Based on Hierarchical Density Estimates*, Berlin, Heidelberg, Springer Berlin Heidelberg: Berlin, Heidelberg, 2013; pp 160-172; (b) McInnes, L.; Healy, J. In *Accelerated Hierarchical Density Based Clustering*, 2017 IEEE International Conference on Data Mining Workshops (ICDMW), 18-21 Nov. 2017; 2017; pp 33-42.
2. Heikkilä, E.; Gurtovenko, A. A.; Martinez-Seara, H.; Häkkinen, H.; Vattulainen, I.; Akola, J., Atomistic Simulations of Functional Au144(SR)60 Gold Nanoparticles in Aqueous Environment. *J. Phys. Chem. C* **2012,** *116*, 9805-9815.
3. Bolintineanu, D. S.; Lane, J. M. D.; Grest, G. S., Effects of Functional Groups and Ionization on the Structure of Alkanethiol-Coated Gold Nanoparticles. *Langmuir* **2014,** *30*, 11075-11085.
4. Koch, A. H. R.; Lévêque, G.; Harms, S.; Jaskiewicz, K.; Bernhardt, M.; Henkel, A.; Sönnichsen, C.; Landfester, K.; Fytas, G., Surface Asymmetry of Coated Spherical Nanoparticles. *Nano Lett.* **2014,** *14*, 4138-4144.
5. Giri, A. K.; Spohr, E., Conformational Equilibria of Organic Adsorbates on Nanostructures in Aqueous Solution: MD Simulations. *J. Phys. Chem. C* **2015,** *119*, 25566-25575.
6. Giri, A. K.; Spohr, E., Influence of Chain Length and Branching on the Structure of Functionalized Gold Nanoparticles. *J. Phys. Chem. C* **2018,** *122*, 26739-26747.
7. Chew, A. K.; Van Lehn, R. C., Effect of Core Morphology on the Structural Asymmetry of Alkanethiol Monolayer-Protected Gold Nanoparticles. *J. Phys. Chem. C* **2018,** *122*, 26288-26297.
8. Kister, T.; Monego, D.; Mulvaney, P.; Widmer-Cooper, A.; Kraus, T., Colloidal Stability of Apolar Nanoparticles: The Role of Particle Size and Ligand Shell Structure. *ACS Nano* **2018,** *12*, 5969-5977.
9. Wu, M.; Vartanian, A. M.; Chong, G.; Pandiakumar, A. K.; Hamers, R. J.; Hernandez, R.; Murphy, C. J., Solution NMR Analysis of Ligand Environment in Quaternary Ammonium-Terminated Self-Assembled Monolayers on Gold Nanoparticles: The Effect of Surface Curvature and Ligand Structure. *J. Am. Chem. Soc.* **2019,** *141*, 4316-4327.
10. Gabrielli, L.; Rosa-Gastaldo, D.; Salvia, M.-V.; Springhetti, S.; Rastrelli, F.; Mancin, F., Detection and Identification of Designer Drugs by Nanoparticle-based NMR Chemosensing. *Chem. Sci.* **2018**, 9, 4777-4784.

11. Wang, E.; Sun, H.; Wang, J.; Wang, Z.; Liu, H.; Zhang, J. Z. H.; Hou, T., End-Point Binding Free Energy Calculation with MM/PBSA and MM/GBSA: Strategies and Applications in Drug Design. *Chem. Rev.* **2019,** *119*, 9478-9508.
12. Wang, C.; Greene, D. A.; Xiao, L.; Qi, R.; Luo, R., Recent Developments and Applications of the MMPBSA Method. *Front. Mol. Biosci.* **2018,** *4*, 87.
13. Homeyer, N.; Gohlke, H., Free Energy Calculations by the Molecular Mechanics Poisson−Boltzmann Surface Area Method. *Mol. Inform.* **2012,** *31*, 114-122.
14. Elder, R. M.; Jayaraman, A., Molecular Simulations of Polycation–DNA Binding Exploring the Effect of Peptide Chemistry and Sequence in Nuclear Localization Sequence Based Polycations. *J. Phys. Chem. B* **2013,** *117*, 11988-11999.
